# High-risk population and factors of stroke has changed among middle-aged and elderly Chinese—Evidence from 1989 to 2015

**DOI:** 10.3389/fpubh.2023.1090298

**Published:** 2023-03-03

**Authors:** Xue Zhang, Jing Dai, Wei Li, Yunjuan Yang

**Affiliations:** ^1^Faculty of Management and Economics, Kunming University of Science and Technology, Kunming, China; ^2^Department of Party Committee, The First People's Hospital of Yunnan Province, Kunming, China; ^3^Yunnan Provincial Center for Disease Control and Prevention, Kunming, China

**Keywords:** stroke, high-risk population, factors, transition, elderly

## Abstract

**Background:**

Stroke is an acute cerebrovascular disease with high mortality and disability. This study aimed to investigate the trend of stroke prevalence from 1989 to 2015 in China, explore the transition of high-risk population and high-risk factors, and provide some evidence to develop more targeted stroke intervention strategies.

**Material and methods:**

We derived the baseline data from China Health and Nutrition Survey (CHNS). Participants responded to face-to-face interviews and examinations containing demographic information, behavioral health information, disease history, and physical examination. We applied chi-square test, shapley value decomposition model, and decision tree model to evaluate the changes of high-risk population and high-risk factors of stroke.

**Results:**

Across 42,419 middle-aged and elderly residents, the prevalence of stroke was decreasing from 1989 to 2015. Hypertension was the leading risk factor of stroke, while its contribution rate was weakened with the increasing of medicine taking rate. As the second risk factor of stroke, the contribution of age decreased either. Meanwhile, the contribution rate of historical health factors, lifestyle factors, and regional factors, such as body mass index, diabetes, and living area to the impact of stroke was increasing. In addition, the first high-risk population of stroke changed from hypertension patients aged 75 years and above to without spouse residents living in stroke belt such as Beijing and Liaoning. The second risk population of stroke transformed from male hypertensive patients under 75 years old into male hypertensive patients living in urban. The third high-risk group turned from the elderly aged 75 and above into the female patients with hypertension and diabetes.

**Conclusions:**

This study demonstrated that the high-risk population and high-risk factors of stroke changed in China and revealed the direction and internal mechanism of transition of stroke. Targeted stroke intervention strategies should be renewed. Health education for the high-risk population of stroke should be carried out, healthy living habits need be advocated, and the use of antihypertensive drugs for the hypertensive patients should be standardized.

## Introduction

Stroke is the second leading cause of death and disability in the world and the global burden of stroke is of continual major importance for global health (1). The prevalence of stroke in European countries and United States ranged from 1.5% in Italy to 3% in the UK and United States (2–4). In Asian countries, the prevalence of stroke has been reported in the range of 45–471/100,000 (5, 6). According to global burden of disease study (GBD) in 2019, stroke is the first cause of life expectancy loss in China (7). The incidence of stroke in China decreased from 222/100,000 in 2005 to 201/100,000 in 2019, and the incidence of ischemic stroke increased from 117/100,000 in 2005 to 145/100,000 in 2019 (8, 9). China is the largest developing country with about one-fifth of the world's population and the highest number of stroke cases in the world. A recent survey conducted by the Stroke Screening and Prevention Committee demonstrated that, the number of new stroke patients in China increased by 8.79% every year and the rate of increase was accelerating. According to China's National Stroke Screening data in 2019, middle-aged and elderly people were more likely to suffer from stroke. In 40 years old and above, the prevalence of first stroke increased from 1.89% in 2012 to 2.58% in 2019 (10).

Overtime, public policies have been established to promote the prevention and treatment of stroke. As of December 2019, the Brain Prevention Committee of the National Health Commission has licensed 30 demonstration senior stroke centers, 466 senior stroke centers, 181 comprehensive stroke prevention centers, and 717 stroke prevention centers (11). The prevention and treatment of cardiovascular and cerebrovascular diseases was integrated into the Healthy China Action in 2019 (12). As an emergency resource (13, 14), the construction of green channels for stroke in hospitals has been improved to shorten the time for stroke patients to be treated. Even so, the prognosis of stroke is still not optimistic. Most survivors have sequelae such as motor disorder, cognitive disorder, and speech swallowing disorder to varying degrees (15), which seriously hurt the self-care ability and mental health of patients, and burden families. Therefore, the prevention of stroke is far more essential than the treatment.

While numerous studies on the etiologies, risk factors, diagnosis, and management of stroke have been conducted in recent decades, it is still one of the main contributors to the burden of disease worldwide (16). In particularly, China is a country with rapid economic growth in recent years, the lifestyle and living standard of residents have modified. The following question is whether the risk factors of stroke, such as hypertension, diabetes, sleep duration, and alcohol, etc. have changed with the lifestyle changes and social progress? Does the high-risk population of stroke change? Is it necessary to determine new screening objects? Thus, Studying the high-risk factors of stroke in China and identifying the changes of high-risk groups has important practical significance for the prevention of stroke and reducing the burden of disease in China. The identification of high-risk population and factors of stroke can also contribute to developing a prevention and treatment roadmap for other non-communicable diseases in the world (17). Considering the importance of this issue this qualitative study described the high-risk factors influencing stroke prevalence and screened the high-risk population of stroke in the middle-aged and elderly in China between the years 1989 and 2015, and aimed to provide national strategies for the prevention and screening of these conditions.

## Materials and methods

**Figure 1:**
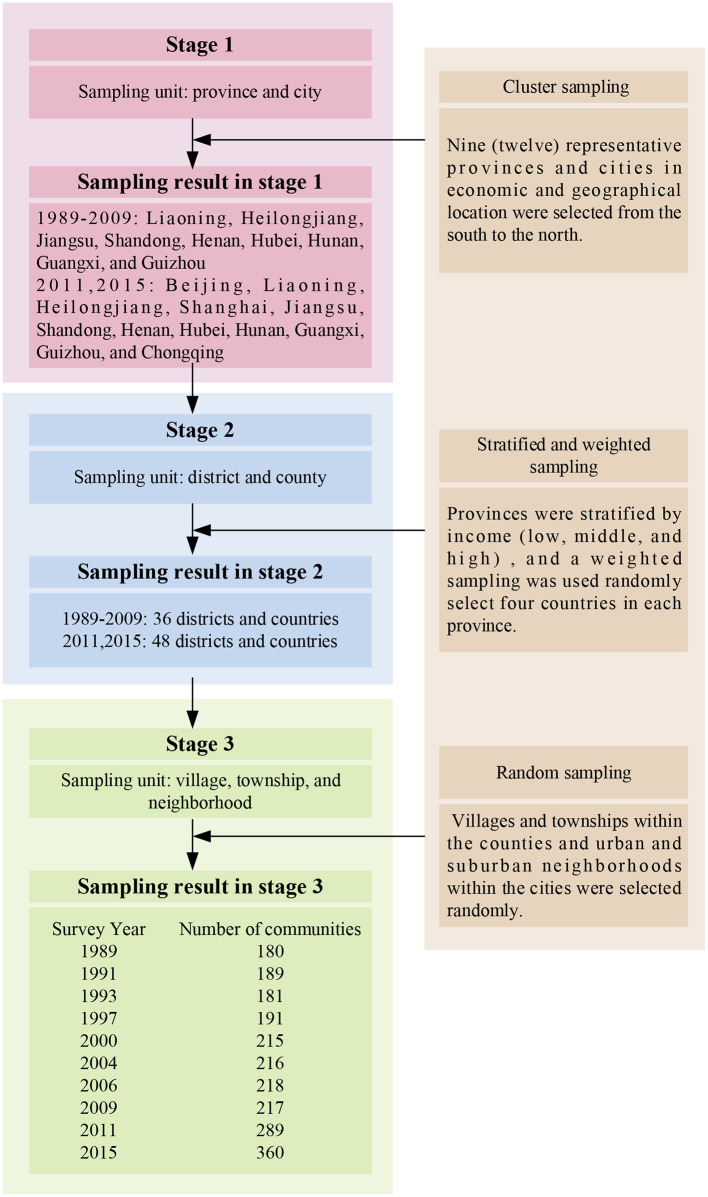
Multistage random cluster sampling process.

The China Health and Nutrition Survey (CHNS) study is an ongoing open cohort international collaborative project between the Carolina Population Center at the University of North Carolina at Chapel Hill and the National Institute for Nutrition and Health at the Chinese Center for Disease Control and Prevention. CHNS offers freely accessible detailed nutrition and health datasets on a sample drawn from China that is representative of the national population. The survey covers nine (twelve) provinces that vary substantially in geography, economic development, public resources, and health indicators. A multistage, random cluster process was used to draw the samples. A detailed sampling process is presented in Figure 1.

### Data collection

**Figure 2:**
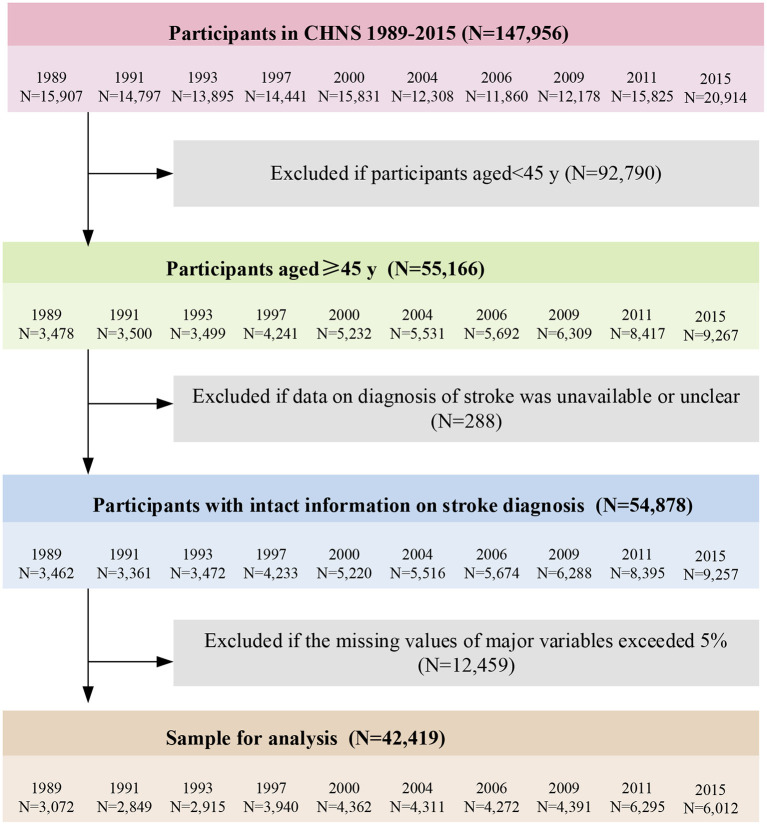
Flow chart.

Participants were contacted and visited at their own homes initially to recruit them into the study. The face-to-face interviews were conducted between trained field staffs and each participant (or his/ her spouse). A series of physical and cognitive measurements was recorded, and a standard questionnaire such as basic demography, health status, nutrition and diet status were answered in each interview. The inclusion criteria in the study were as follows: (1) Age 45 years and above. (2) Should have clear diagnosis of stroke. (3) Should have completed (missing value < 5%) major variables data. Then, we excluded participants as follow: age below 45 years (*N* = 92,790), with unavailable or unclear stroke diagnosis (*N* = 288), with missing value of marital status and educational attainment (*N* = 2,380), occupation and individual income (*N* = 3,077), smoking and alcohol behavior (*N* = 2,471), activity participation and sleep duration (*N* = 2,542), hypertension, diabetes and atrial fibrillation (*N* = 621), as well as height and weight (*N* = 1,368). The detailed sample selection process is presented in Figure 2. This study was approved by the Ethics Review Committee of The First People's Hospital of Yunnan Province. Written informed consent was obtained from all participants.

### Stroke definition

Participants were asked “Did your doctor give you a diagnosis of stroke or transient ischemic attack?” in the face-to-face interviews. Response options included “Yes,” “No,” and “Not clear.” If participants responded “Yes,” they would be defined as having incident stroke outcomes. If participants answered “No,” they would be classified as having no incident stroke outcomes. Participants with “Not clear” answer were excluded from this study.

### Potential risk factors

This study covered four dimensions of risk factors including sociodemographic factors, lifestyle factors, historical health variables, and regional information. The sociodemographic factors taken into account were gender, age, residents, marital status, educational attainment, logarithm of individual income, and occupation types. Lifestyle factors included smoking, Alcohol, activity participation, and sleep duration. Smoking and drinking were defined as a self-reported history in last year. Of the six self-reported activities (martial art, track, gymnastics, soccer or basketball, badminton or volleyball, and ping pong), participating in one or more was defined as participation activity. Sleep duration was divided three levels according self-reported sleep time. Historical health variables included hypertension, diabetes, atrial fibrillation, and body mass index (BMI). Hypertension was measured using mean values of three systolic and diastolic blood pressures. According to the latest standards of WHO, systolic blood pressure ≥140 *mmHg* and diastolic blood pressure ≥90 *mmHg* were defined as hypertension. Diabetes and atrial fibrillation were defined as a self-reported physician diagnosis. BMI was calculated by dividing weight by height squared. Height and weight were measured with participants wearing light clothing and without shoes during the physical examination. Regional information referred to the province where the respondent was located.

### Statistical analysis

Chi-square tests were used to examine the general characteristics differences between stroke and non-stroke subjects according to the distribution of categorized variables. Count and percentages were used to describe categorical variables. The risk factors, which were significant in the chi-square test, were further analyzed by decision tree model, which was established by Chi-squared Automatic Interaction Detection (CHAID). Shapley value decomposition model was adopted to calculate the contribution rate and ranking of high-risk factors. *P* < 0.05 was considered statistically significant. The decision tree analysis method is an analysis tool based on the principle of probability theory (18). Its basic principle is to use decision points to represent decision problems (19). Compared with the traditional logic regression method, the decision tree analysis method can directly reflect the characteristics of different subgroups and the proportion of different results, and can also reflect the interaction between variables (20). It is mostly used for prediction analysis and factor analysis. However, the disadvantage of this method is that cannot reflect the quantitative impact of variables. The combination of decision tree analysis and Shapley Value Decomposition can just make up for their shortcomings.

## Results

### Characteristics of all participants

Table 1 shows the distribution of the main characteristics of the study population. A total of 42,419 middle-aged and elderly residents aged 45 and above were included in the survey from 1989 to 2015. The distribution of male and female was uniform, accounting for nearly 50%, respectively. Among them, the middle-aged residents aged 45–49, 50–54, and 55–59 were the most. Most residents lived in rural areas, while only 37.5% residents lived in cities and towns. Moreover, the 82.1% of residents had spouses. The proportion of illiterate residents was 41.56%, and the proportion of residents with high school education or above was < 20%. More than half of the residents had an annual income of < 5,000 yuan. In terms of the type of occupation, the number of residents without jobs was the largest, accounting for 42.92%, followed by farmers, accounting for 31.57%. In terms of lifestyle, most residents maintained good living habits, no smoke, no drink, and kept normal sleep duration. However, the exercise time and amount of most residents were below the standard (89.53%). In terms of historical health information, 18.52% of residents suffered hypertension, 4.34% suffered diabetes, 1.29% suffered heart disease, and more than 30% kept a BMI of 25 kg/m^2^ or above. The residents participating in the study were spread across 12 provinces in China, among which Guangxi Province and Jiangsu Province had the largest number, accounting for 11.64 and 11.09%, respectively.

**Table 1:** General characteristics of middle-aged and older Chinese ≥45, 1989–2015.

**Item**	**Total**		**Normal**		**Stroke**		***χ^2^***	***P*-value**
	**No**.	**%**	**No**.	**%**	**No**.	**%**		
Total	42,419	100.00	41,384	97.56	1,035	2.44		
**Gender**							43.478	< 0.001
Female	21,425	50.51	21,007	98.05	418	1.95		
Male	20,994	49.49	20,377	97.06	617	2.94		
**Age groups (years)**							368.663	< 0.001
45–49	8,624	20.33	8,553	99.18	71	0.82		
50–54	8,283	19.53	8,168	98.61	115	1.39		
55–59	7,516	17.72	7,378	98.16	138	1.84		
60–64	6,443	15.19	6,244	96.91	199	3.09		
65–69	4,742	11.18	4,568	96.33	174	3.67		
70–74	3,339	7.87	3,177	95.15	162	4.85		
75–79	2,068	4.88	1,962	94.87	106	5.13		
80 above	1,404	3.31	1,334	95.01	70	4.99		
**Residents**	42,419						72.379	< 0.001
Rural	26,512	62.50	25,996	98.05	516	1.95		
Urban	15,907	37.50	15,388	96.74	519	3.26		
**Marital status**								
No spouse	7,592	17.90	7,342	96.71	250	3.29	28.265	0.001
Having spouse	34,827	82.10	34,042	97.75	785	2.25		
**Educational attainment**								
Illiterate	17,631	41.56	17,100	96.99	531	3.01	50.858	< 0.001
Elementary school	8,905	20.99	8,719	97.91	186	2.09		
Middle school	8,754	20.64	8,587	98.09	167	1.91		
High school	5,719	13.48	5,613	98.15	106	1.85		
College or more	1,410	3.32	1,365	96.81	45	3.19		
**Individual income (RMB)**							13.235	0.004
Q1 (< 1,244)	10,604	25.00	10,298	97.11	306	2.89		
Q2 (1,244–4,456.89)	10,602	24.99	10,351	97.63	251	2.37		
Q3 (4,456.89–13,800)	10,584	24.95	10,357	97.86	227	2.14		
Q4 (>13,800)	10,629	25.06	10,378	97.64	251	2.36		
**Occupation types**							128.702	< 0.001
No jobs	18,205	42.92	17,584	96.59	621	3.41		
Professional and administrator	4,006	9.44	3,949	98.58	57	1.42		
Worker	5,636	13.29	5,536	98.23	100	1.77		
Farmer	13,390	31.57	13,158	98.27	232	1.73		
Rests	1,182	2.79	1,157	97.88	25	2.12		
**Smoking**								
No smoking	28,313	66.75	27,637	97.61	676	2.39	0.980	0.322
Smoking	14,106	33.25	13,747	97.45	359	2.55		
**Alcohol**								
No alcohol	28,961	68.27	28,146	97.19	815	2.81	53.6926	< 0.001
Alcohol	13,458	31.73	13,238	98.37	220	1.63		
**Activity participation**								
No activity	37,977	89.53	37,043	97.54	934	2.46	0.576	0.448
Activity	4,442	10.47	4,341	97.73	101	2.27		
**Sleep duration (h/d)**								
< 6	1,935	4.56	1,884	97.36	51	2.64	129.603	< 0.001
6−10	39,346	92.76	38,448	97.72	898	2.28		
>10	1,138	2.68	1,052	92.44	86	7.56		
**Hypertension**								
No hypertension	34,565	81.48	34,216	98.99	349	1.01	16.013	< 0.001
Hypertension	7,854	18.52	7,168	91.27	686	8.73		
**Diabetes**								
No diabetes	40,576	95.66	39,684	97.80	892	2.20	229.007	< 0.001
Diabetes	1,843	4.34	1,700	92.24	143	7.76		
**Atrial fibrillation**								
No atrial fibrillation	41,873	98.71	40,900	97.68	973	2.32	184.691	< 0.001
Atrial fibrillation	546	1.29	484	88.64	62	11.36		
**BMI (kg/m** ^**2**^ **)**								
< 25	28,062	66.15	27,502	98.00	560	2.00	102.381	< 0.001
25–30	10,313	24.31	10,020	97.16	293	2.84		
30 above	4,044	9.53	3,862	95.50	182	4.50		
**Provinces**							95.258	< 0.001
Beijing	1,070	2.52	1,029	96.17	41	3.83		
Liaoning	3,603	8.49	3,478	96.53	125	3.47		
Heilongjiang	3,521	8.30	3,447	97.90	74	2.10		
Shanghai	1,616	3.81	1,562	96.66	54	3.34		
Jiangsu	4,705	11.09	4,595	97.66	110	2.34		
Shandong	4,384	10.33	4,269	97.38	115	2.62		
Henan	4,460	10.51	4,321	96.88	139	3.12		
Hubei	4,219	9.95	4,110	97.42	109	2.58		
Hunan	4,128	9.73	4,052	98.16	76	1.84		
Guangxi	4,939	11.64	4,809	97.37	130	2.63		
Guizhou	4,631	10.92	4,576	98.81	55	1.19		
Chongqing	1,143	2.69	1,136	99.39	7	0.61		

### Prevalence of stroke in different populations

As shown in Table 1, gender, age, residents, marital status, personal income, occupation, drinking, hypertension, sleep duration, diabetes, heart disease, BMI, and provinces were the main influencing factors for stroke among middle-aged and elderly residents in China (*P* < 0.05). Among them, middle-aged and elderly men were more likely to suffer from stroke compared with female middle-aged and elderly residents. Compared with rural residents, urban residents were more likely to suffer from stroke. The middle-aged and elderly residents with spouse were less likely to suffer from stroke than those without spouse. What's more, the prevalence of stroke increased with age. The residents with the lowest and highest education levels were more likely to suffer from stroke. The lower the individual's annual income, the higher the possibility of stroke. Moreover, the unemployed middle-aged and elderly residents were more likely to suffer from stroke. The middle-aged and elderly residents who did not drink alcohol were more likely to suffer from stroke than those who drank alcohol. Both excessive sleep and insufficient sleep could promote the onset of stroke. Besides, the prevalence of stroke in middle-aged and elderly residents with hypertension, diabetes, and heart disease was higher than that in normal residents. In addition, the larger the BMI, the more likely to suffer from stroke. What's more, the prevalence of stroke in Liaoning, Beijing, Shanghai, and Henan was the highest, all exceeding 3%.

### High-risk factors and high-risk population of stroke

**Figure 3:**
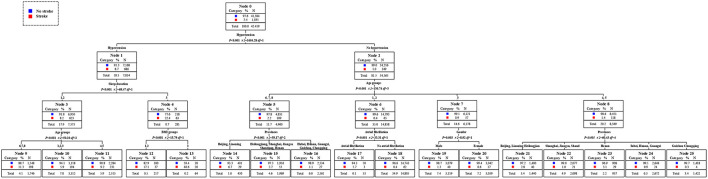
Assessment of risk factors and the susceptible population of stroke, 1989–2015.

According to the results of the decision tree in Figure 3, hypertension was the primary factor affecting the prevalence of stroke, followed by sleep time and age. BMI, province, heart disease, and gender were the third level risk factors affecting the prevalence of stroke in middle-aged and elderly residents. From the left branch of the decision tree, the prevalence of stroke in middle-aged and elderly residents with hypertension alone was 8.7%. Moreover, the prevalence of stroke in middle-aged and elderly residents with hypertension and more than 10 h of sleep per day reached to 22.4%, becoming the second high-risk group. The middle-aged and elderly residents with hypertension, sleeping more than 10 h per day, and the BMI of more than 30 kg/m^2^ were the first risk group for stroke, and the risk of stroke reached 40.6%. Otherwise, the elderly residents with hypertension, sleeping within 6–10 h a day, and over 70 years old were the third high-risk group, and the prevalence rate of stroke was 11.3%. From the right branch of the decision tree, residents who did not suffer from hypertension, under the age of 70–84, and lived in Beijing and Liaoning, becoming the fourth high-risk group for stroke, with a prevalence rate of 6.7%. The fifth high-risk group was middle-aged residents aged 45–54 who did not suffer hypertension but suffered heart disease, and the prevalence rate of stroke was 5.7%. Additionally, the prevalence of stroke among middle-aged residents aged between 45 and 60 who did not suffer from hypertension or heart disease was below the average level. The prevalence of stroke was also below the average level for residents who did not suffer from hypertension, 60–69 years old, and living in Shanghai, Jiangsu, Shandong, Hubei, Hunan, Guangxi, Guizhou, and Chongqing.

### Changes trends of stroke prevalence in high-risk factors

**Figure 4:**
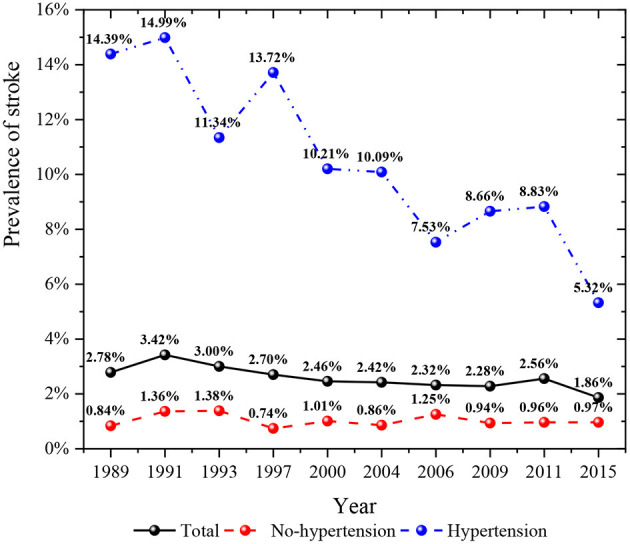
Prevalence of stroke in hypertension and no-hypertension groups in 1989–2015.

**Figure 5:**
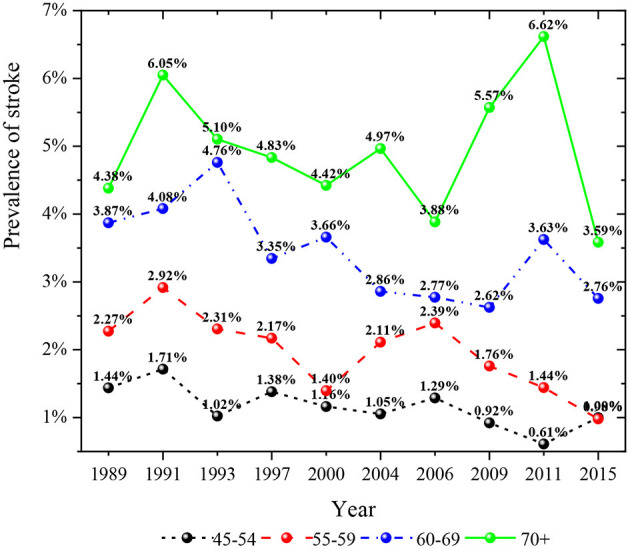
Prevalence of stroke in different age groups in 1989–2015.

**Figure 6:**
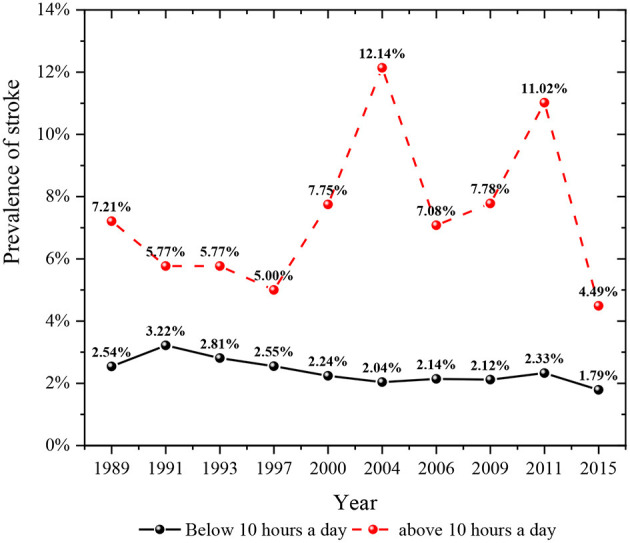
Prevalence of stroke in different sleep duration groups in 1989–2015.

As shown in Figure 4, the prevalence of stroke in the general population decreased from 1989 to 2015. The prevalence of stroke in people with hypertension was higher than that in people without hypertension. In recent years, the prevalence of stroke among middle-aged and elderly residents who did not suffer from hypertension had an upward trend. However, the prevalence of stroke among middle-aged and elderly residents suffering from hypertension had shown a rapid decline trend in recent years, especially by 2015, the prevalence of stroke among hypertensive people had dropped to the lowest in history. Figure 5 described the change of the prevalence of stroke among middle-aged and elderly residents of all ages after regrouping by classification decision tree. The prevalence of stroke among middle-aged residents aged 45–54 years showed a downward trend. The prevalence of stroke in residents aged 55–59 years was higher than that in residents aged 45–54 years at the same period, and the prevalence of stroke in residents aged 55–59 years also showed a downward trend. Moreover, the prevalence of stroke among residents aged 60–69 years showed a downward trend. The prevalence of stroke among residents aged 70 years and above fluctuated greatly in different periods, while it was in a downward trend. It could be seen from Figure 6 that the prevalence and change trend of stroke among middle-aged and elderly residents with different sleep duration after regrouping by classification decision tree. The prevalence of stroke among middle-aged and elderly residents who slept < 10 h a day was at a low level and in the process of slow decline. By 2015, the prevalence rate was the lowest. Otherwise, the prevalence of stroke among middle-aged and elderly residents who slept more than 10 h a day fluctuated greatly, while the prevalence of stroke was generally in a downward trend.

Table 2 showed the contribution of stroke influencing factors to stroke prevalence in middle-aged and elderly residents. Hypertension was the first risk factor affecting stroke among middle-aged and elderly residents in 2009, 2011, and 2015, but its contribution was gradually declining, with the contribution of 50.91% in 2009, 44.51% in 2011 and 36.39% in 2015. Age was always the second major risk factor for stroke in middle-aged and elderly residents. And the contribution rate of age to the prevalence of stroke was also in a downward trend, from 19.78 to 11.01%. Hypertension, age, sex, residence, and occupation type contributed the most to the prevalence of stroke among middle-aged and elderly residents in 2009, with a total contribution of 80.43%. In addition, hypertension, age, occupational type, province, and diabetes were the main risk factors affecting stroke among middle-aged and elderly residents in 2011, with a total contribution of 88.01%. Compared with 2009, the impact of historical health factors on stroke in middle-aged and elderly people was increasingly prominent. Hypertension, age, province, diabetes, and sleep duration were the main risk factors affecting stroke in middle-aged and elderly people in 2015, with a total contribution of 76.92%. Besides, compared with 2009, the contribution of historical health factors and behavioral style factors to stroke had gradually increased.

**Table 2:** Shapley value decomposition results of risk factors of stroke.

**Item**	**2009**		**2011**		**2015**	
	**Contribution rate (%)**	**Rank**	**Contribution rate (%)**	**Rank**	**Contribution rate (%)**	**Rank**
Gender	5.31	3	4.12	6	6.52	7
Age	19.78	2	23.26	2	11.01	2
Residents	4.43	4	0.48	11	2.51	9
Marital status	0.32	14	0.50	10	2.94	8
Educational attainment	1.19	12	0.37	13	0.84	11
Individual income	0.73	13	0.10	14	0.52	12
Occupation types	3.51	5	9.92	3	7.75	6
Alcohol	2.24	9	0.88	9	0.19	14
Sleep duration	2.89	6	3.38	7	9.55	5
Hypertension	50.91	1	44.51	1	36.39	1
Diabetes	2.77	7	4.95	5	9.68	4
Atrial fibrillation	1.85	10	1.72	8	1.35	10
BMI	2.36	8	0.44	12	0.47	13
Provinces	1.71	11	5.37	4	10.29	3
Total	100.00	—	100.00	—	100.00	—

### Changes trends of stroke prevalence in high-risk population

**Figure 7:**
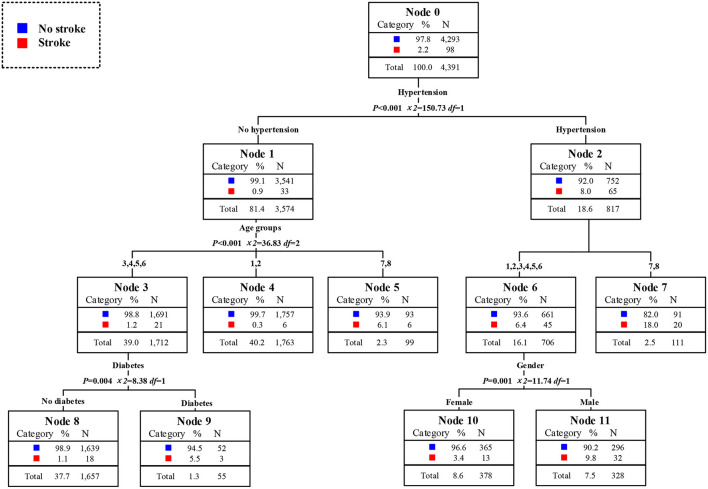
Assessment of risk factors and the susceptible population of stroke in 2009.

**Figure 8:**
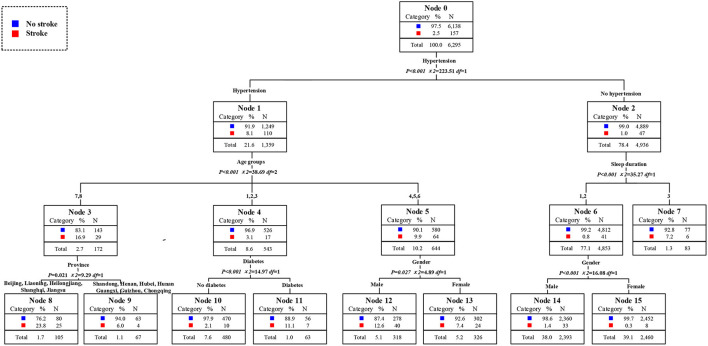
Assessment of risk factors and the susceptible population of stroke in 2011.

**Figure 9:**
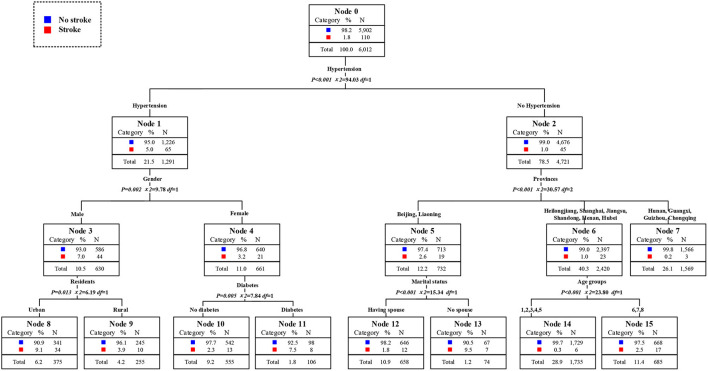
Assessment of risk factors and the susceptible population of stroke in 2015.

The high-risk population of stroke in 2009 could be identified from the classified statistical results in Figure 7. The prevalence rate of stroke among middle-aged and elderly people was 2.2% in 2009. The prevalence rate of stroke among residents with hypertension was 8.0%, which was 8.9 times than that of residents without hypertension. Among hypertension patients, the prevalence rate of stroke in patients aged 75 years and above was as high as 18.0%, that was, the elderly hypertensive patients aged 75 years and above were at high risk of stroke. The prevalence of stroke in men aged 45 years and above and under 75 years who also suffered from hypertension was 9.8%, which was also a high-risk population. The second was the 55 to 74 years old elderly who did not suffer from hypertension but had diabetes. The prevalence rate of stroke was 5.5%, 2.5 times higher than that of ordinary middle-aged and elderly people. In conclusion, the elderly with hypertension was the first high-risk group for stroke in 2009. Middle aged men and young elderly people with hypertension were the second high-risk group for stroke. The elderly who did not suffer from hypertension but were only over 75 years old were the third high-risk group for stroke. The young elderly without hypertension but with diabetes were the fourth high-risk group for stroke. It could be concluded that age and historical health problems were the main factors to divide the high-risk population of stroke. The distribution of high-risk population of stroke in 2011 could be obtained from Figure 8. The overall prevalence of stroke among middle-aged and elderly residents was 2.5% in 2011. The prevalence of stroke in patients with hypertension was 8.1%, which was 8.1 times higher than that in residents without hypertension. In particular, the prevalence rates of stroke among the elderly over 60 years old and over 75 years old were 9.9 and 16.9%. At the same time, the prevalence of stroke was 23.8% in elderly who suffered from hypertension and lived in Beijing, Shanghai, Jiangsu, Liaoning, Heilongjiang, which either in high level of economic development or northeast areas. They were the first high-risk population. Meanwhile, the prevalence of stroke among hypertensive patients with diabetes aged between 45 and 60 years was 11.1%. The prevalence rate of stroke in male hypertensive patients aged 60–74 was 12.6%, which was five times higher than that in ordinary middle-aged and elderly residents. For residents who did not suffer from hypertension, excessive sleep time had become the first killer of stroke. For residents who slept more than 10 h a day, the prevalence of stroke was 7.2%. In a word, the elderly hypertensive patients who lived in Beijing, Shanghai, Jiangsu, and Northeast China in 2011 were the first risk group for stroke, and the male hypertensive patients aged 60–74 years were the second risk group for stroke. Middle aged residents with diabetes and hypertension were the third risk group for stroke. What's more, middle-aged and elderly residents who slept more than 10 h a day were also at high risk of stroke. In conclude, age and historical health problems were no longer the main risk groups of stroke, regional factors, and healthy life factors had also became the key factors to divide the high-risk groups. As shown in Figure 9, the prevalence of stroke among middle-aged and elderly residents in China was 1.8% in 2015.. The prevalence of stroke in patients with hypertension was 5.0%, which was five times higher than that in patients without hypertension. In addition, the prevalence of stroke in male hypertensive patients living in cities and towns was higher, which was 9.1%. The prevalence of stroke among middle-aged and elderly female residents with diabetes and hypertension was 7.5%, 4.2 times higher than that of ordinary middle-aged and elderly residents. Meanwhile, among the people who did not suffer from hypertension, the prevalence of stroke among the elderly who lived in Beijing, and Liaoning and without spouse was 9.5%. In a word, unmatched middle-aged and elderly residents living in Beijing and Liaoning were the first high-risk group for stroke in 2015. Male hypertensive patients living in cities and towns were the second risk group for stroke. The middle-aged and elderly female residents with diabetes and hypertension were the third high-risk group for stroke. It could be seen that the high-risk group of stroke had not only been limited to patients with historical health problems such as hypertension and diabetes, but also started to extend to middle-aged and elderly people without spouse.

## Discussion

**Figure 10:**
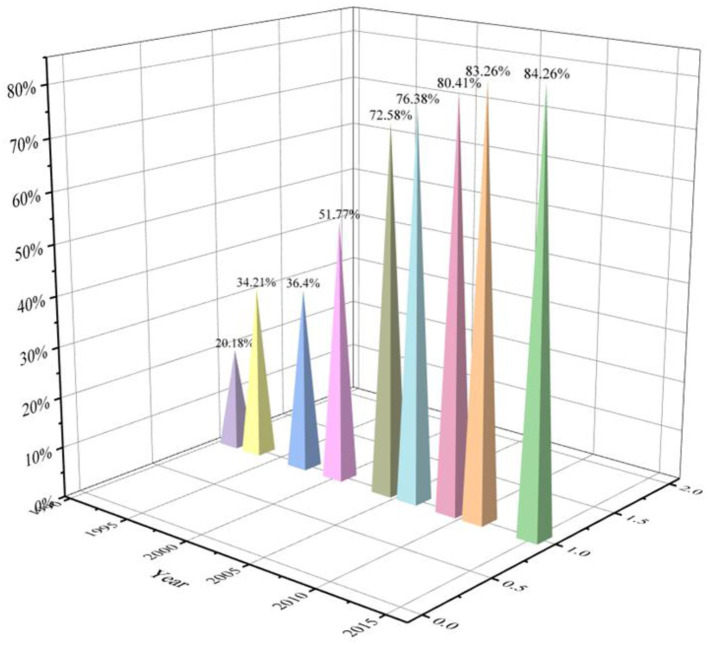
Medicine taking rate of hypertension in 1991–2015.

This study was the first to evaluated the transition of high-risk factors and high-risk population of stroke in middle-aged and older Chinese from 1989 to 2015. It has provided some useful information for stroke prevention and intervention in middle-aged and older Chinese. Hypertension was the first risk factor of stroke in China, but in recent years, the influence of hypertension was gradually decreasing. Hypertension could lead to remodeling of cardiovascular and cerebral vessels, damage the function of pressure reflex, induce white matter lesions, and then lead to stroke (21). In addition, pulmonary hypertension will cause the hemoglobin in the blood to be too viscous, which will lead to blockage of blood vessels, thus causing stroke (22). However, hypertension has been shown to be the single most important modifiable risk factor in adult stroke (23). Hypertension patients taking drugs to control hypertension could effectively reduce the prevalence of stroke among residents in the Stroke Belt (24). The drug taking rate and control rate of hypertension were gradually rising in China. It could be seen from Figure 10 of this study that the treatment rate of hypertension in China had increased from 20.18 to 84.26% from 1991 to 2015. Therefore, with the control rate of hypertension increased, the impact of hypertension on stroke also decreased (25, 26). Age was the second risk factor of stroke in China, but its influence was gradually weakening in recent years. With the increase of age, patients' arterial elasticity decreased, and intima damage was more likely to occur, which promoted lipid deposition to form plaque, leading to stroke (27). However, with the improvement of medical level and the improvement of life expectancy in China, the impact of age on stroke was on the decline. Reasonable diet, scientific exercise and regular monitoring could largely compensate for the disease risk brought by aging to the elderly (28–30). Short or long sleep time would increase the risk of stroke in middle-aged and elderly people, and the contribution rate of sleep time to stroke in elderly people was increasing year by year. The existing clinical observation results showed that insufficient and excessive sleep duration could directly or indirectly cause stroke by influencing endocrine and metabolic functions, sympathetic nerve excitability, cortisol level and other mechanisms, such as hypertension, diabetes, and dyslipidemia (31, 32). In addition, people at high risk of stroke were generally accompanied by hypertension, diabetes, hyperlipidemia, obesity and other diseases, and some cerebrovascular functions were damaged. The cerebral blood flow and cerebral metabolism of such people would change significantly with the change of sleeping habits (33). Hence, people with insufficient and excessive sleep duration suffered a higher stroke risk.

With the progress and development of Chinese society, the high-risk factors of stroke among middle-aged and elderly residents in China had also changed accordingly. Changed from unmodified factors such as age, gender, and residence to behavior factors such as occupation type and sleep duration. Previous studies had shown that there were significant occupational differences in stroke. The service industry, agriculture, forestry, animal husbandry and fishery had higher stroke risk than other occupations (34). Moreover, these occupations had a higher detection rate of cardiovascular risk factors than other occupations, including poor blood glucose control, and obesity (35). Targeted workplace interventions could help reduce cardiovascular risk factors in these occupational groups, thereby benefiting health. In addition, compared with residents without jobs, engaging occupations can promote and help sustain healthy lifestyle habits for person for cardiovascular diseases, including stroke (36). In addition, even those had high generic risk but adhering to healthy lifestyle had a lower risk of stroke than those at low-to-intermediate genetic risk of stroke (37). This further confirmed the importance of a healthy lifestyle in reducing stroke risk.

The high-risk population of stroke had changed from the elderly residents with historical health problems to the residents living in certain areas and adhering to certain lifestyles. Stroke Belts had been formed in Beijing, Shanghai, Liaoning, Heilongjiang, and Henan in China. Middle-aged and elderly people living in these regions had a higher risk of stroke. On the one hand, studies showed that the regional differences were due to the different levels of physical activity of residents in each region. Adult Stroke Belt residents had markedly lower physical activity levels and were less likely to meet physical activity guidelines than their non-Stroke Belt counterparts (38). The work pressure in Beijing and Shanghai was high, and the time was tight, especially the middle-aged residents in the career development period were seriously lacking in physical activity level. On the other hand, air pollution was a new modifiable neuro-vascular risk factor (39). One study reported the risk for ischemic stroke was increased after exposure to air pollution and exposure to air pollution increased the risk of intracerebral hemorrhage (40). Notably Beijing was one of the most polluted capitals in the world and the average air quality index (AQI) in 2016 was slight pollution (41). In fact, Shanghai was a mega city in China, while its AQI was classified as “good” on only 58 and 78 days in 2017 and 2016 (42). What's more, it was confirmed that solid fuel use for cooking and heating associated with increased risk of stroke, while persistent clean fuel use for both heating and cooking associated with lower risk of stroke occurrence (43). However, Liaoning, Heilongjiang, and Henan were major agricultural production provinces in China, straw burning and solid fuel using was common before the revision of the law on prevention and control of air pollution in 2016. Hence, the stroke risk in China may be closely connected with air pollution in living area. Notably, the impact of living area on stroke was obvious in the classification tree statistics in 2011 and 2015. The possible reason was that Chongqing, Beijing, and Shanghai were included in 2011 and 2015. If these three cities were included in the first wave of survey, more obvious results may appear. Because it can be seen in the shapley value analysis that the contribution rate of living area was growing, and the ranking is getting top. Our study has highlighted the changing role of hypertension and the stronger role of behavior, lifestyle, and living area. In addition, health education is also necessary (44). Combine the stroke screening with the health examination of residents, carry out health education for the high-risk population of stroke, advocate healthy living habits, and standardize the use of antihypertensive drugs for the hypertensive patients. On this basis, health files of stroke should be built at the community level, and combined the stroke health management with the Chinese family doctor contract system, regularly follow up the stroke high-risk population.

Notwithstanding, this study has certain limitations. First, the diagnosis of stroke relied on self-reporting and, despite face-to-face interviews, there were inevitable misreported. Second, this study lacked specific distinction between ischemic and hemorrhagic stroke, as more than 50% of the cases cannot report their stroke type clearly. Third, the participants across the ten waves of survey lacked coherence for many participants exited or new entered. Accuracy of the trends may be undermined because generation gap and period effect.

## Conclusion

High-risk population and factors of stroke had changed among middle-aged and older Chinese from 1989 to 2015. Hypertension and age had less influence on stroke, while diabetes, sleep duration and marital status had greater influence on stroke. High risk population of stroke had changed from residents with historical health problems and old age to residents living in certain areas and adhering to certain lifestyles. Furthermore, if possible, we can subdivide the types of stroke in future study, and explore the changes of high-risk factors and high-risk population of hemorrhagic stroke and ischemic stroke, respectively.

## Data availability statement

Publicly available datasets were analyzed in this study. This data can be found here: https://www.cpc.unc.edu/projects/china China Health and Nutrition Survey.

## Ethics statement

The study was approved by the Ethics Review Committee of the First People's Hospital of Yunnan Province. The patients/participants provided their written informed consent to participate in this study.

## Author contributions

XZ statistical analysis and manuscript preparation. JD study concept and design. WL study supervision and critical revision of manuscript for intellectual content. YY statistical analysis and interpretation of data. All authors contributed to the article and approved the submitted version.
